# Factors Determining Quarantine in Infectious Disease

**Published:** 1916-04

**Authors:** Geo. L. Nicholas

**Affiliations:** Chief of Division of Epidemiology, Dept. of Health, City of New York


### Factors Determining Quarantine in Infectious Disease.

By DR. GEO. L. NICHOLAS,

Chief of Division of Epidemiology, Dept. of Health, City of New York.

Dismissal from quarantine is not in any case based solely upon an arbitrary time limit. Minimum periods of quarantine, fixed for certain diseases, are to a large extent determined by bacteriological investigation and animal experimentation even where the specific germ is not actually known, for example in measles, where reducing the minimum quarantine to five days from the appearance of the rash, appears to be justified by recent studies in which the blood was found uniformly to have lost evidence of infectivity about the second day after the appearance of the rash. Further, aside from the laboratory study, we determine fitness for discharge from quarantine by the disappearance of fever, freedom from crusts or scabs, and the absence of catarrhal and suppurative processes.

The "carrier" is looming larger and larger as the active agent in the dissemination of a number of communicable diseases. Especially is this true of the convalescent or so-called acute carrier. Therefore, one might almost feel justified in taking the word in its real meaning and ask for an actual **quarantine,** a term of forty days. In fact, this is done in the cases of poliomyelitis.. Such a measure would not by any means cover the whole situation. Chronic carriers who may or may not have had the disease themselves are another great danger. The handling of carriers is a part of quarantine work scarcely yet touched upon. Some of the reportable diseases listed in the New York City Sanitary Code are not officially quarantinable, that is, under pressure of other circumstances, the Board of Health does not regard them as sufficiently serious or readily communicable as to demand an enforced quarantine. In such diseases, however, isolation is often desirable. Here moral considerations come in. The ethics of infection is a topic not much dwelt upon, yet it is along these lines developed by popular education and awakened conscience, that any real success in preventing certain communicable diseases must come.

Take typhoid, for instance. How long shall we quarantine the patient? When a person recovered from typhoid comes to know the danger from carriers, and the limitations of the best laboratory work, if he is an honest man he will never discharge himself from a proper measure of isolation, never lift his quarantine. This does not mean that he cannot go about his daily affairs, but he will exercise such care and decency as to protect others from germs he may perchance excrete. For the individual sick with any communicable disease, isolation in due measure, if only behind a handkerchief when he sneezes, must be taught. It is his duty to keep this up in adequate degree until absolutely sure that all danger is passed.

Diphtheria is the only disease (ordinarily occurring in New York) that is regularly discharged from quarantine upon bacteriological findings, two consecutive negative cultures from both nose and throat being required. Here is a case exactly to the point. It was found in following the above routine that a good many cases slipped through at a too early date, the two negative cultures being succeeded by a positive culture at the next trial. On this account, a definite time limit was added, not altogether arbitrary, but based upon the observation that a large percentage of diphtheria cases were free from bacilli in about two weeks after the onset. The rule of two consecutive negative cultures remains, but cultures for discharge may not be taken before the twelfth day after report of the case. This prevents discharge of the patient before the fourteenth day of the disease at the earliest (see Weekly Bulletin of February 19, 1916, page 59).

Dealing with diphtheria cases in which the bacilli persist is another matter. Here a virulence test is made which, if negative, permits release of the patient.

Scarlet Fever, on the other hand, is a disease in which quarantine is most nearly arbitrary, the minimum being thirty days from the appearance of the rash. Desquamation is not regarded as of any special significance as to infectivity; suppurative processes are more significant and ordinarily serve to confine the patient for several weeks longer. This arbitrary period is not always efficient in preventing secondary cases. Investigation of a series of scarlet fever patients discharged from Department hospitals after thirty-five days, and to all appearances quite free from the disease, showed that in some two per cent of the families involved secondary cases promptly developed among the other children. The present time limit of scarlet fever quarantine does not seem to be excessive.

In Measles the patient may be freed from isolation and allowed to return to school, for instance, five days after the appearance of the rash. The quarantine of exposed persons who have not had the disease, so far as school is concerned, is maintained at the old period of fourteen days. This early closing of measles cases is confined to those who are free from serious catarrhal or pneumonic complications. The rule has been in force now three years, and there is no evidence that any increase in measles cases has resulted. As noted above there is a hint of bacteriological backing for this new procedure in measles. The matter of isolation in measles is left in the hands of the private physician, a postal card certificate being filled in by the attendant for presentation at school.

In Typhoid Fever there is no time limit. In all questions of quarantine the laboratory report is relied on. There is a tendency to be much more particular about isolation in this disease. By a new regulation of the Board of Health now lately put in force (see Weekly Bulletin January 22, 1916, page 30) no typhoid cases may be discharged until feces and urine taken not later than ten days after temperature has become normal have been submitted for bacteriological examination. Except in cases of food handlers, a positive result does not keep the individual from going about his affairs, but, of course, with instructions as to care about infecting others. A food handler may not resume work after typhoid fever until cleared by the laboratory. The only difficulty that may arise in carrying out this method is in the magnitude of the work when the disease is especially prevalent. Collecting stool specimens is not simple. Examination must be started promptly on the day the stool is passed. An extra heavy influx of specimens might well swamp a laboratory. Under such circumstances it would be better to confine the work to a thorough examination of food handlers and others with important social relations and let the rest go. While a single negative stool in the early days after typhoid fever is not conclusive, a positive result is very impressive to the patient and his friends. It is strongly educative. The Department of Health under some circumstances forcibly removes chronic typhoid carriers. All are kept under supervision as far as possible.

In Smallpox the patient is removed to the Department of Health hospital, and strictly isolated until free from all scabs, including the deep seated ''seeds'' beneath the epidermis of the soles of the feet. Suppurative processes should have subsided. There is no time limit. Exposed persons are kept under observation and in some instances in quarantine for twenty-one days.

For Epidemic Cerebro-spinal Meningitis the time is fixed at fourteen days. It has been found that meningococci in the naso-pharynx of convalescents are very rare after the twelfth or the fourteenth day. On the other hand, the relatively very large number of non-clinical carriers always occurring in an outbreak of this disease make quarantining a discouraging matter. There is no bacteriological examination for discharge.

For acute Poliomyelitis anterior the minimum isolation period is six weeks. This, too, is in a sense fixed by bacteriological consideration. It is clear that carriers exist, and the time limit is made as long as possible in reason.

In Yellow Fever, infectivity has been shown to disappear early in the clinical course. This has weight in deciding how long the patient must remain in quarantine—quarantine here meaning simply isolation from the mosquito.

In Epidemic Typhus Fever, strict isolation of the patient in the Department of Health hospital until the fever has entirely subsided is required. In former years the practice was to supply all patients on discharge with an entirely new outfit of wearing apparel, old clothing and bedding being destroyed. There was also very thorough sulphur fumigation at the patient's home. At that time the way in which the disease is communicated was not known, but the above routine was exactly suited to the actual facts. Exposed persons were regularly held in quarantine for twenty-one days. There has been no occasion to handle Epidemic Typhus in the light of recent bacteriological studies.

In Brills' disease (modified or Endemic Typhus) sulphur fumigation is done at the patient's home. With due precautions as to lice the patient need not be further isolated. Recovery is marked by the subsidence of the fever.

For Cholera, the termination of the quarantine of the patient is strictly bacteriological. A minimum requirement is two consecutive negative stools taken at intervals of five days after convalescence is complete. Carriers occur. Exposed persons are quarantined five days.

After recovery from plague a patient presenting any suppurative or ulcerating lesion would be subjected to examination for the bacillus pestis, certainly before allowing him to go to a new locality.

In the cases of such diseases as Smallpox, Epidemic Typhus, Cholera and Plague, general principles alone can be given. In the handling of an actual outbreak of one of these diseases procedure would be modified according to the circumstances.

In the minor so-called contagious diseases, as German Measles, Mumps, Chicken Pox and Whooping Cough, isolation is not so strictly maintained. In German Measles it is arbitrarily fixed at seven days, in Mumps until all swelling is gone, in Chicken Pox until all scabs are off, while in Whooping Cough some more definite routine is being developed. It is believed that the more infectious period is in the early days. The specific organisms are scarcely found after the second week. Stricter isolation is therefore relaxed two weeks after the onset of the spasmodic cough. But this is an instance for relative isolation and recognition of personal responsibility. When taken out, the child must be kept strictly away from other and susceptible children until the spasmodic cough has entirely gone.

In Pulmonary Tuberculosis stricter isolation, even forcible removal to the hospital, is often required. Here again, bacteriological considerations determine the question of allowing the patient greater freedom. The moral obligation of the tuberculous subject is to be pressed to the utmost. If individual isolation by all recognized precautions is not maintained by the patient, he is liable to removal to a sanatorium.

